# Cysteine-cysteine chemokine receptor 5 (CCR5) profile of HIV-infected subjects attending University of Calabar Teaching Hospital, Calabar, Southern Nigeria

**DOI:** 10.1186/s12879-019-4737-1

**Published:** 2020-01-03

**Authors:** Ekerette Friday Ekere, Monday F. Useh, Henshaw Uchechi Okoroiwu, Tatfeng Youtchou Mirabeau

**Affiliations:** 1 Hematology Laboratory, University of Calabar Teaching Hospital, Calabar, Nigeria; 2 Microbiology Unit, Department of Medical Laboratory Science, University of Calabar, Calabar, Nigeria; 3 Hematology Unit, Department of Medical Laboratory Science, University of Calabar, Calabar, Nigeria; 4 Department of Medical Laboratory Science, College of Health Sciences, Niger Delta University, Amassama, Nigeria

**Keywords:** CCR5, HIV, CCR5-Δ32, CCR5 genotype, Mutant CCR5, Wild type CCR5

## Abstract

**Background:**

Cysteine-cysteine chemokine receptor 5 is the main HIV co-receptor involved in the virus and cell-to-cell spread. A variant of the CCR5 gene known as CCR5-Δ32 which is a product of 32 base pair deletion in the gene plays critical role in the infection and progression to AIDS. The study was carried out to determine the CCR5 genotype of HIV-infected subjects attending University of Calabar Teaching Hospital, Calabar.

**Methods:**

A total of 100 subjects attending HIV clinic, University of Calabar Teaching Hospital were purposively recruited for this study. DNA was extracted from each sample using the Quick gDNA miniprep DNA extraction kit, Zymo Research. Polymerase chain reaction (PCR) was used in the amplification of CCR5 gene in each DNA in a 9700 ABI Thermo cycler and then resolved on 4% agarose gel electrophoresis.

**Result:**

Out of the 100 samples assessed, 100 (100%) were homozygous for the CCR5 wild type gene (CCR5-wt), while none (0%) was homozygous for the CCR5-Δ32 (mutant type), and heterozygosity was not observed.

**Conclusion:**

This study observed absence of CCR5-Δ32 deletion gene among the studied subjects in Calabar, implying lack of genetic advantage in HIV infection and possible rapid progression towards AIDS if other precautions are not checked.

## Background

Human immunodeficiency virus (HIV)/ Acquired immunodeficiency syndrome (AIDS) despite the campaigns has remained a public health concern [1] with approximately 36.7 million people living with HIV globally at the end of 2016. The burden of the epidemic varies between regions and countries with sub-Saharan Africa being the most affected having nearly 1 in every 25 adult living with HIV accounting for two-third of people living with HIV globally [2–4]. According to the 2014 Gap report, 9% of those living with HIV reside in Nigeria [5]. With 3.2 million (9%) people living with HIV in Nigeria as of 2016, she has ranked the second largest HIV global disease burden behind South Africa that housed 7.1 million (19%) people living with HIV [6, 7]. Infection with HIV is associated with progressive loss of cellular immunity which results to life threatening opportunistic infections and progressive development of acquired immunodeficiency syndrome [8].

The major event in HIV infection is the continuous decrease in CD4^+^T cells that leads to deficient immune system that is unable to withstand the actions of opportunistic pathogens. The more interesting part is the significant disparity of individuals in the susceptibility to the infection, the time lag for depletion of CD4^+^ T – lymphocytes, as well as time lag to progress to AIDS-defining state [9–11]. Host genes collectively known as AIDS restriction genes (ARGs); C-C type chemokine receptor 5 (CCR5) and C-X-C chemokine receptor type 4 (CXCR4) have been known to modify individual response to HIV-1 exposure, infection and pathogenesis markedly [12–14]. The human C-C type chemokine receptor (CCR5) belongs to the G-protein-coupled receptor family and is found mostly on the cell surface of some white blood cells such as T4 cells, monocytes and macrophages [15, 16]. The CCR5 wild type protein (CCR5-wt) is made up of 352 amino acids folded into 7 membrane-spanning domains linked by 3 intracellular loops. Aside the wild type, there is CCR5 allele with a 32 base pair deletion (CCR5-Δ32) in the coding region. This mutation results in a truncated protein that cannot be detected on the cell surface [14, 15]. The CCR5-Δ32 mutation has been shown to significantly affect the entry of the HIV and also the progression to AIDS [14]. Individuals who are homozygous for this defect (polymorphism) appear to be resistant to HIV-1 infection [12, 14] and same retards disease progression in heterozygotes [17]. HIV gains entry into the host’s cell through its glycoprotein 120 receptor (gp120) which binds to the hosts cell receptor cluster of differentiation four (CD4^+^) on T lymphocytes. For the virus to enter the host cell, it requires membrane fusion. This occurs through the virus glycoprotein 41 (gp41) which catalyzes the fusion of the virus to a host co -receptor being either CCR5 or CXCR4, and anchors the virus to the host membrance [17]. In the case of the mutated protein (in this case CCR5-Δ32) prevents the fusion of the virus to the host’s cell surface, thereby denying if entry into the cell, hence, rendering the cell resistant to HIV [12, 17]. The R5-tropic virus strain of HIV-1 prevail using CCR5 as co-receptor for fusion with the cell in the early stage of infection [18–20], while the X4-tropic strain of HIV-1 utilizes CXCR4 as co-receptor [21–23]. Probably due to the emergence of the X4-tropic HIV strains, the protective effect of the homozygous deletion in the CCR5 gene is not entire [24]. The CCR5- *∆* 32 mutatiion is predominantly found in European population and rare or no occurrence in the Asians and African population [12, 14, 25].

Considering the lack of information of CCR5 profile in Calabar, Cross River State, this study set off to bridge the gap in literature.

## Method

### Study location

**Fig. 1:**
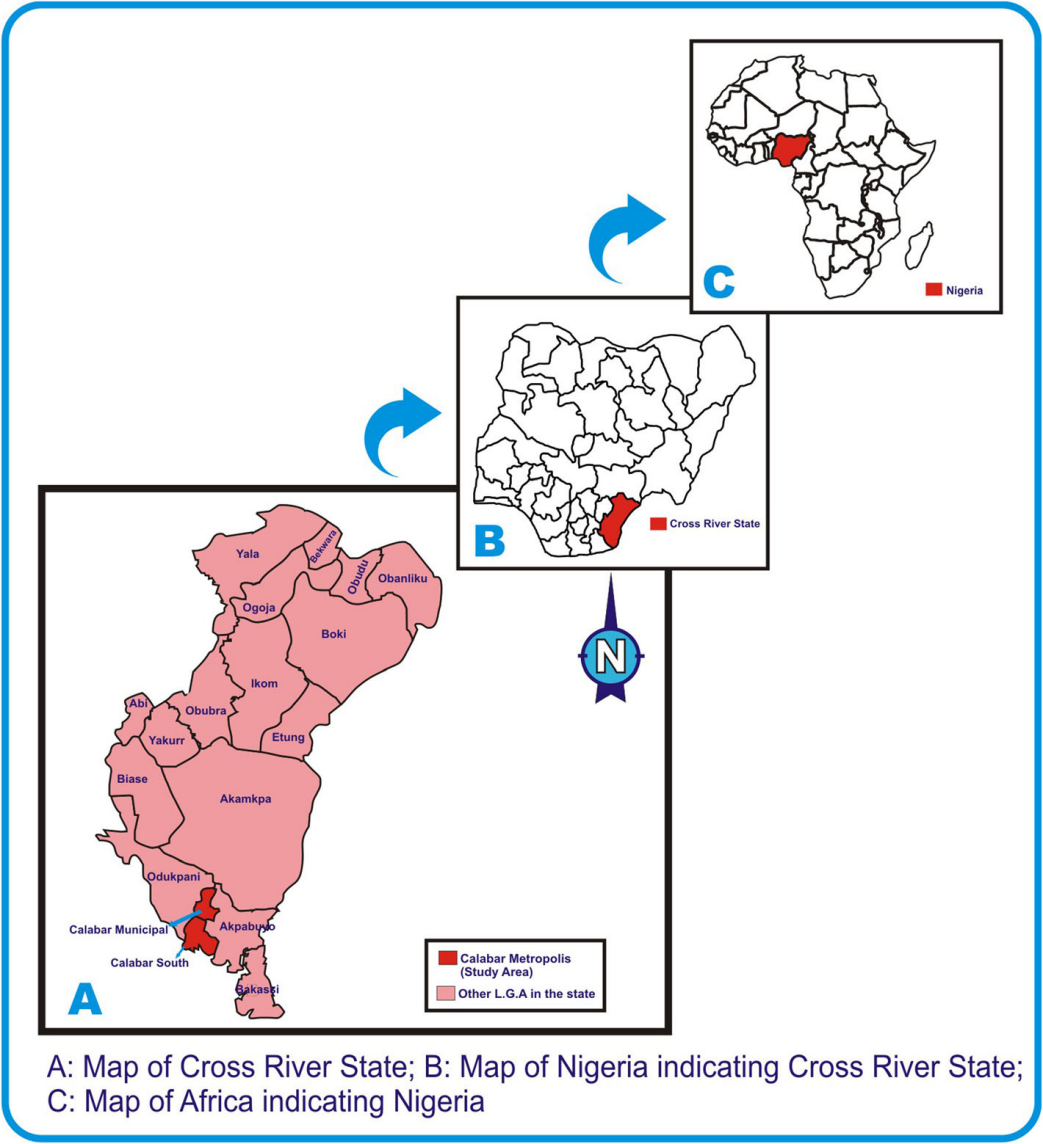
Map of study area

This cross sectional study was carried out in University of Calabar Teaching Hospital, Cross River State, Nigeria. Cross River State is located in Southern Nigeria [26] in the Niger Delta Region. It is bounded in the north by Benue State, the west by Ebonyi and Abia State, the east by Cameroon Republic and the south by Akwa Ibom State and the Atlantic Ocean [27]. Cross River State has an area of 21,787km^2^ and a population of 2,892,988 (2006 census) [28]. University of Calabar is sited in Calabar Metropolis which is a fusion of Calabar south local government and Calabar municipality. Calabar has a geographical coordinates of 8^0^19′37.02E with an estimated population of 375,196 (2006 census) [28, 29]. The map showing the study area is in indicated in Fig. 1.

### Study population

One hundred HIV-infected clients attending antiretroviral therapy (ART) clinic of University of Calabar Teaching Hospital were purposively recruited for this study. Positive subjects who have been confirmed and were on ART treatment who gave their consent were recruited. HIV negative and HIV positive subject who declined were excluded.

### DNA extraction

DNA was extracted using gDNA mini prep DNA extraction kit, Zymo Research supplied by Inqaba Biotechnological South Africa. One hundred microliter (100 μL) of whole blood was pipetted into a 1.5 ml. Four hundred microliters (400 μL) of the genomic lysis buffer were added. The samples were mixed by votexing for 5 s and were allowed to stand at room temperature for 10 min. The mixtures were transferred to a zymo-spin column in a collection tube. It was then centrifuged at 12,000 rpm for 1 min. The flow through and collection tubes were discarded. The zymo-spin columns were transferred to a new collection tube and 200 μL of DNA pre-wash buffer was added and centrifuged at 12,000 rpm for 1 min. Five hundred microliter (500 μL) of gDNA was buffered and was added to the spin column and centrifuged at 12,000 rpm for 1 min. The spin column was then transferred into another micro-centrifuge tube and 50 μL of DNA elution buffer added to the spin column and incubated at room temperature for 5 min followed by centrifugation for 30 s to elute the DNA. The eluted DNA was then used for PCR.

### CCR5 gene amplification

The CCR5 gene was amplified by polymerase chain reaction (PCR) utilizing primers that flank the 32-base pair deletion; primary: P1: P1:5^’^CAAAAGGTCTTCATTACACC3′ and secondary: P2: P2:5’CCTGTGCCTCTTCTCATTTC-3’ on a 9700 ABI thermal cycler. The PCR mixture included 1X master mix (which contains Tq polymerase, DNTPS, MgCl_2_), forward and reverse primers at concentration of 0.4 M and 3 μL of the extracted DNA. DNAse free water was used to make up the PCR to a final volume of 20 μL. The PCR conditions used for the amplification of the gene was as follows: 5 cycles of 94 °C for 3 min (initial denaturation), 94 °C for 45 s (denaturation), 55 °C for 45 s (annealing), 72 °C for 1.5 s (extension) and and a final extension of 72 °C for 5 min and 30 cycles at 94 °C for 30 s (denaturation), 60 °C for 30 s (annealing) and 72 °C for 30 s (extension) and a final extension of 72 °C for 3 min. A 5 μl aliquot of each amplicon were resolved on 4% agarose gel at 120 V for 20 min and visualized in an ultraviolet trans-illuminator. The presence of a single fragment of 185 bp was considered to represent homozygote for CCR5 gene.

### HIV-V3 region amplification

A two-step PCR amplification, first with outer primers and then with nested or inner primers, was performed to detect the presence of HIV-1 in infected patients’ PBMC. The DNA oligonucleotide primers HIV-19: 5′-AATGTCAGCACAGTACAATGTACA-3′ and HIV-20: 5′-CAGTAGAAAAAATTCCCCTCCACAATT-3′ and HIV-21: 5′-CTGCTGTTAAATGGCAGTCTAGC-3′, and HIV-22: 5′-TCTGGGTCCCCTCCTGAGGA-3′ were used The PCR components comprised of 5 PCR buffer (100 mM Tris-HCl [pH 8.3], 500 mM KCl, 15 mM MgCl_2_, 0.01% gelatin), 200 mM each dATP, dCTP, dGTP, and TTP, 0.2 to 1.0 mM each HIV-1 outer primer pair. The reactions were carried out at 94 °C for 1.5 min, 45 °C for 2 min, and 72 °C for 3 min for 35 cycles. The amplified DNA products were analyzed by electrophoresis on a 1.2% agarose gel. Negative controls consisting of DNA from PBMC of seronegative individuals were included in each set of reactions, which were negative in all assays. After the first round PCR, 1 ml of the product was amplified for 25 cycles with the corresponding inner primers at 94 °C for 1.5 min, 50 °C for 2 min, and 72 °C for 3 min. The PCR products were analyzed by electrophoresis on a 1.2% agarose gel.

### Sequencing of CCR5 and HIV-V3 gene

The sequencing of the V3 gene and CCR5 was done using the Big Dye Terminator kit on ABI 3500 sequence by Inquaba, South Africa.

### Phylogenic analysis

Obtained sequences were edited using the Bioinformatic software Bioedit. Similar sequences were downloaded from the National Centre for Bioinformatics Information (NCBI) using Blast N. Downloaded sequence were aligned using the alignment software MAFT and the evolutionary relationship was eliminated at 500 bootstrap using MEGA.

## Results

Table 1 shows the virological and clinical details of the studied population. Majority (65%) of the studied subjects were females. Subjects with CD4 < 200 cells/μL, CD4 200–499 cells/μL and CD4 ≥ 500 cells/μL consisted 14, 41 and 45% of the studied population. Majority of the studied subjects fell into the CDC category A. The studied participants weren’t sure of their mode of acquisition of HIV (Table 1).

**Table 1:** Virological and clinical characteristics of the studied subjects

Parameter	Frequency (%)			
CD4 category	Total	A	B	C
CD4 ≥ 500 cells/μL	45 (45)	43 (95.6)	2 (4.4)	0 (0.0)
CD4 200–499 cells/μL	41 (41)	29 (70.7)	9 (22.0)	3 (7.3)
CD4 < 200 cells/μL	14 (14)	4 (28.6)	7 (50.0)	3 (21.4)
ART intake				
On ART	100 (100)			
ART Naïve	0 (100)			
Gender				
Male	35 (35)			
Female	65 (65)			
Mode of acquisition	0 (0)			
Vertical	0 (0)			
Blood transfusion	0 (0)			
Sexual	0 (0)			
Not sure	100 (100)			

Table 2 shows the sequencing of CCR5 receptor protein among the HIV-infected subjects and reveals a similar protein sequence without any mutation among amino acid sequence with high concentration of Leucine 7 (15.99%) followed by Lysine 5 (11.6%).

**Table 2:** Sequence profiling of CCR5 receptor protein

SEQ Name		Protein sequence			
1_R5F_H10_22		FWKNFQTLKI VILGLVLPLL VMVICYSGIL KTLLRCRNEK KRHR			
2_R5F_A11_02		FWKNFQTLKI VILGLVLPLL VMVICYSGIL KTLLRCRNEK KRHR			
3_R5F_B11_05		FWKNFQTLKI VILGLVLPLL VMVICYSGIL KTLLRCRNEK KRHR			
5_R5-F_D05_11		FWKNFQTLKI VILGLVLPLL VMVICYSGIL KTLLRCRNEK KRHR			
7_R5-F_F05_17		FWKNFQTLKI VILGLVLPLL VMVICYSGIL KTLLRCRNEK KRHR			
8_R5-F_G05_20		FWKNFQTLKI VILGLVLPLL VMVICYSGIL KTLLRCRNEK KRHR			
9_R5-F_H05_23		FWKNFQTLKI VILGLVLPLL VMVICYSGIL KTLLRCRNEK KRHR			
10_R5-F_A06_03		FWKNFQTLKI VILGLVLPLL VMVICYSGIL KTLLRCRNEK KRHR			
11_R5-F_B06_06		FWKNFQTLKI VILGLVLPLL VMVICYSGIL KTLLRCRNEK KRHR			
12_R5-F_C06_09		FWKNFQTLKI VILGLVLPLL VMVICYSGIL KTLLRCRNEK KRHR			
13_R5-F_D06_12		FWKNFQTLKI VILGLVLPLL VMVICYSGIL KTLLRCRNEK KRHR			
14_R5-F_E06_15		FWKNFQTLKI VILGLVLPLL VMVICYSGIL KTLLRCRNEK KRHR			
15_R5-F_F06_18		FWKNFQTLKI VILGLVLPLL VMVICYSGIL KTLLRCRNEK KRHR			
16_R5-F_G06_21		FWKNFQTLKI VILGLVLPLL VMVICYSGIL KTLLRCRNEK KRHR			
17_R5-F_H06_24		FWKNFQTLKI VILGLVLPLL VMVICYSGIL KTLLRCRNEK KRHR			
18_R5-F_A07_01		FWKNFQTLKI VILGLVLPLL VMVICYSGIL KTLLRCRNEK KRHR			
19_R5-F_B07_04		FWKNFQTLKI VILGLVLPLL VMVICYSGIL KTLLRCRNEK KRHR			
20_R5-F_C07_07		FWKNFQTLKI VILGLVLPLL VMVICYSGIL KTLLRCRNEK KRHR			
Code	Name	Ratio	Code	Name	Ratio
F	Phenylalanine	2 (4.5%)	I	Isoleucine	4 (9.1%)
W	Tryptophan	1 (2.3%)	V	Valine	3 (6.4%)
K	Lysine	5 (11.4%)	C	Cysteine	2 (4.5%)
N	Asparagine	2 (4.5%)	S	Serine	1 (2.3%)
Q	Glutamine	1 (2.3%)	R	Arginine	4 (9.1%)
T	Threonine	1 (2.3%)	H	Histidine	1 (2.3%)
L	Leucine	7 (15.9%)	P	Proline	1 (2.3%)
M	Methionine	1 (2.3%)	E	Glutamate	1 (2.3%)
G	Glycine	1 (2.3%)			
Y	Tyrosine	1 (2.3%)			

**Fig. 2:**
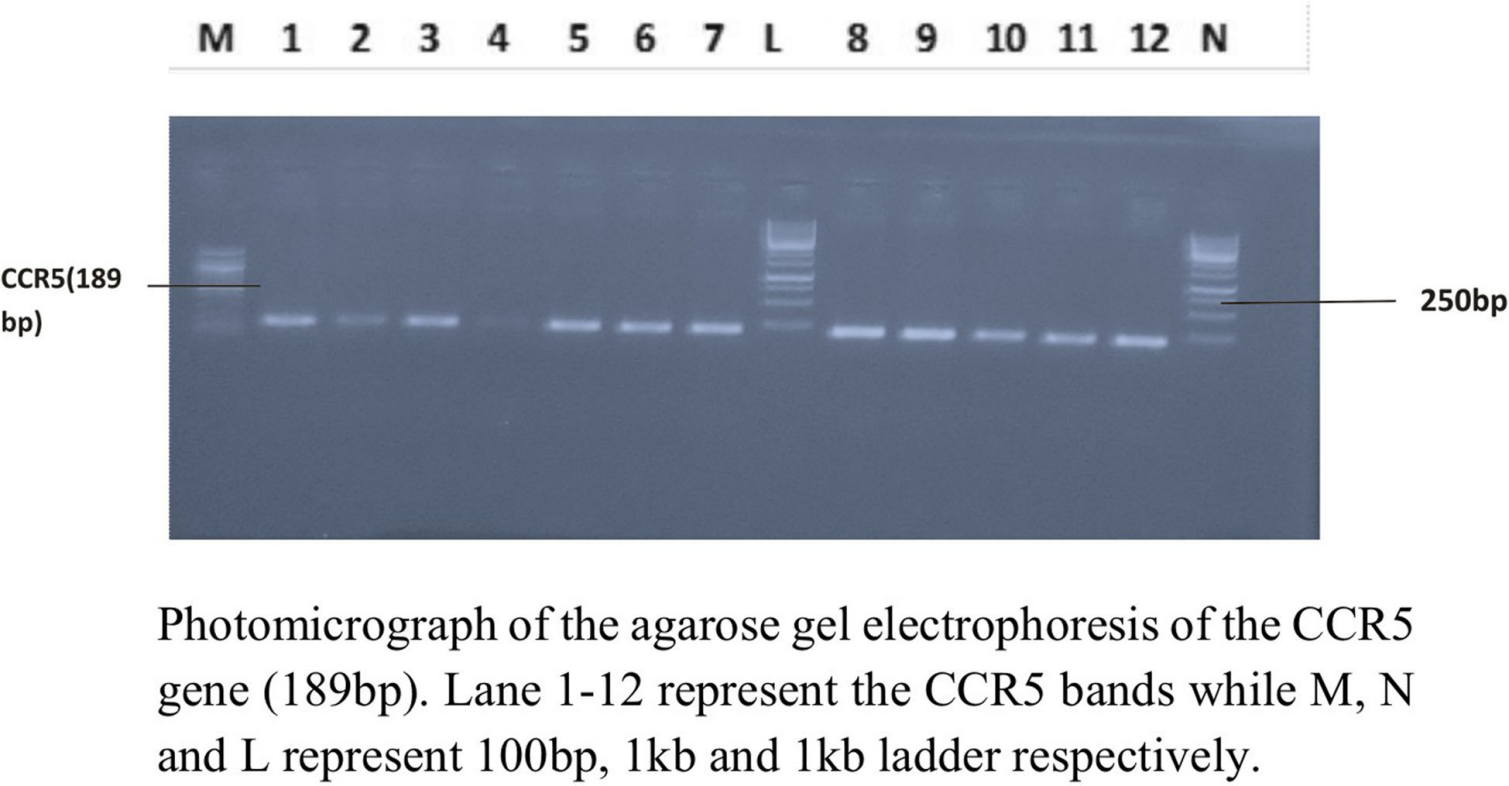
Profile of CCR5 gene among HIV-infected subjects using PCR

Figure 2 shows the profiling of CCR5 gene among the HIV-infected participants using PCR. The amplified CCR5 gene resolved on agarose at 120 V for 20 min showed that CCR5 DNA bands were slightly ahead of 200 bp of the ladder and the sizes were estimated at 189 bp. The gel revealed that all he subjects (100%) were homozygote for CCR5-wt gene, as no band was detected at 157 bp indicating a 32 bp deletion.

Table 3 shows the frequency of CCR5 gene genotype in the studied population. All the assessed subjects (100%) were homozygous for the wild type CCR5 (CCR5-wt). No homozygous mutant type (CCR5-Δ32) nor heterozygote was observed.

**Table 3:** Frequency of CCR5 genotype of the studied HIV-infected population

Gene variant	Frequency	Percentage (%)
Wild type (CCR5-wt)	100	100
Mutant type (CCR5-Δ32)	0	0

**Fig. 3:**
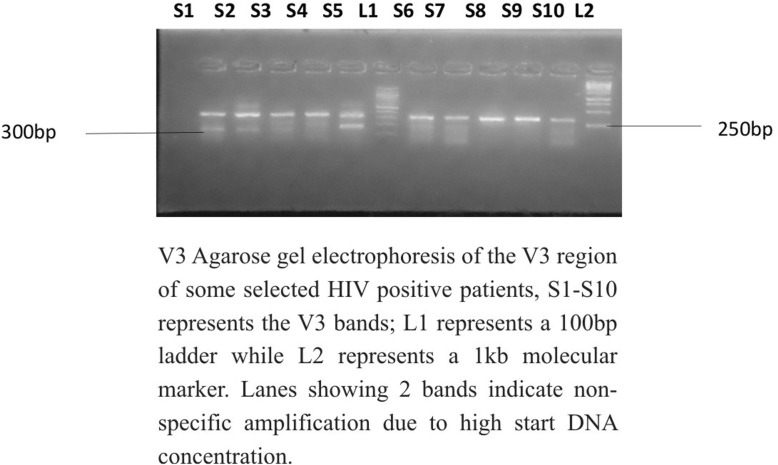
PCR product showing amplified V3 gene

The amplified V3 gene was resolved on agarose at 120 V for 20 min and visualized on UV trans-illuminator. The subjects amplicons were ran along a 100 bp Biolabs molecular ladder. The V3 DNA bands aligned with the 300 bp of the ladder and the sized were estimated to be 300 bp as expected (Fig. 3).

**Fig. 4:**
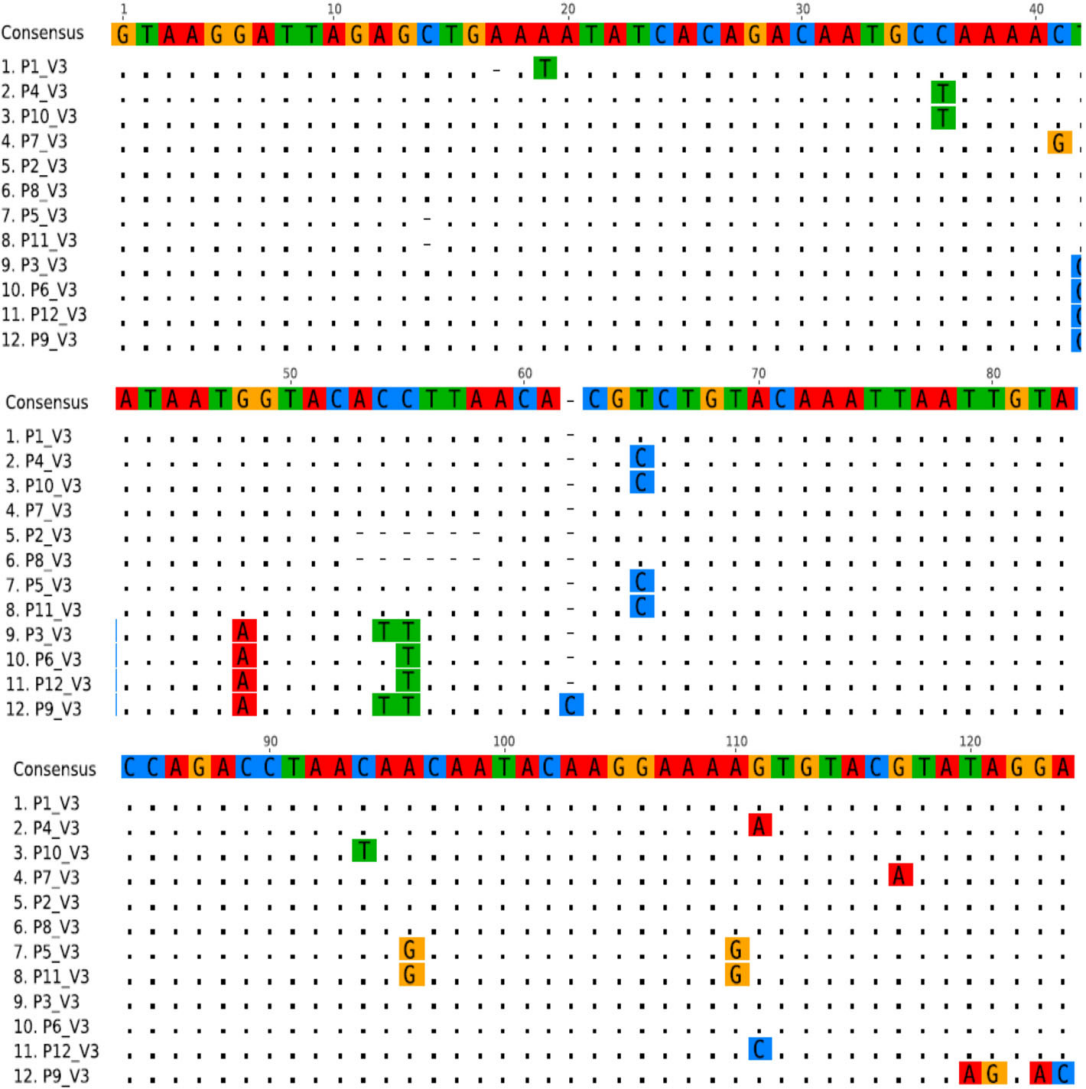
Sequence alignment of the V3 gene showing the various nucleotide polymorphisms

**Fig. 5:**
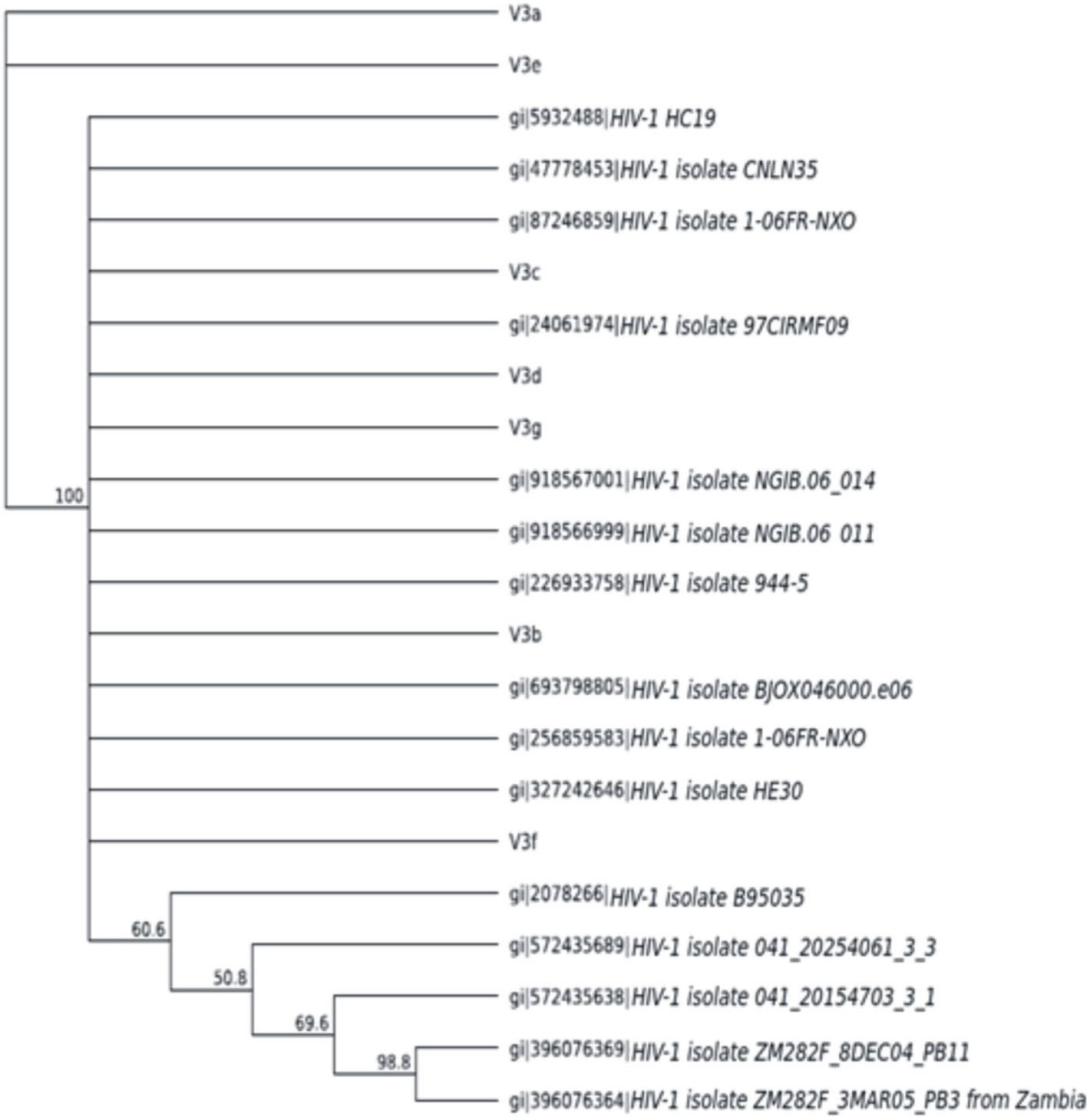
Phylogenic tree showing the genetic relationship between the HIV strains

Figure 4 shows sequence alignment of the V3 gene showing the various nucleotide polymorphisms, while Fig. 5 shows the phylogenic tree showing the genetic relationship between the HIV strains. The obtained V3 sequences from the patients produced an exact match during the megablast search for highly similar sequences from the NCBI non-redundant nucleotide (nr/nt) database. The V3 sequence showed a percentage similarity to other V3 region ranging from 98 to 100%. The evolutionary distances computed using the Jukes-Cantor method were in agreement with the phylogenetic placement of the V3 sequences within the HIV and revealed a closely relatedness to HIV-1 isolate 97CIRMF09 (gi\24,061,974) from Senegal, HIV-1 HC 19 (gi/5932488) from Gabon and HIV-1 (gi/918567001) from Cameroon. Furthermore, the gene2pheno software revealed that all the strains were HIV1 subtypes C (Table 4) though the sequence alignment showed some variations in the nucleotides sequence of the V3 gene of various strains (Fig. 4).

**Table 4:** HIV-1 strains used

Strain	HIV-1 subtype
P1	C
P2	C
P3	C
P4	C
P5	C
P6	C
P7	C
P8	C
P9	C
P10	C
P11	C
P12	C

## Discussion

The CCR5 gene profiling among the HIV-infected subjects in Calabar showed that all (100%) subjects that participated in the study were homozygotes for the normal allele/wild type (CCR5-wt). There was no detection of CCR5 *∆* 32 mutation. The absence of CCR5 *∆* 32 mutation observed in this study is consistent with 1% earlier report by Martinson and colleagues [25] for Nigeria and a more recent report of Nil value by Kenneth et al., 2016 [30] in Bayelsa State, Nigeria. However, higher frequencies of CCR5- *∆* 32 mutation has been reported in northern Europe, particularly the Baltic region as represented by data by Roy and Chakrabarti [9] in Sweden, Belarus, Finland, Lithuania and Estonia. Other areas with reported high frequency of CCR5- *∆* 32 polymorphism included northern coast of France, the Russian cities of Moscow and Ryazan, portions of Volga-Ural region of Russia [31]. Population based studies reported the phenotype of the wild type gene as 75.56% in Rusia [32], 87.5% in Turkey [25], 98.21% in Cyprus Greek [32], 91.22% in Jordan [32], 97.16% in Syria [32], 97.96% in Kuwait [31], 100% in Yemen [25] being studies in Euro - Asia while 100% has been reported in Kenya [25] and Sudan [33]. The relatively higher frequency of the wild type gene of the CCR5 in African region is suggestive that the mutant variant (CCR5- *∆* 32) is fairly recent in terms of human evolution [34, 35].

The implication of the absence of the CCR5- *∆* 32 gene in the studied population revealed a poor resistance to HIV and above all, underlined the fact that our HIV infected subjects are likely rapid progressors. The V3 sequencing showed genetic relationship between HIV strains isolated in this study and those isolated from Senegal P3764 (V3e), Garbon (V3d and Veg), V3b from Cameroon (V3b) and (V3e) from Senegal, this relatedness among strains could be due to trans-border movement of the population which may facilitate the transfer of one stain from a region to the other,

## Conclusion

This study has revealed the absence of the mutant gene type of CCR5 (CCR5-Δ32) in the studied subjects implying that the studied population lack one of the prominent genetic advantage against HIV-infection as well as possible rapid progression to AIDS, hence the need to strengthen management and diagnostic strategies.

## Data Availability

Datasets generated and analysed in this study are available from the corresponding author on request.

## Abbreviations

AIDS: Acquired immunodeficiency syndrome
ART: Antiretroviral therapy
CCR5: C-C- type chemokine receptor
CD: Cluster of differentiation
CXCR4: C-X-C chemokine receptor 4
DNA: Deoxyrebonucleic acid
HIV: Human Immunodeficiency virus
PCR: Polymerase chain reaction
